# Genetic deletion of hepatic NCOR1 protects from atherosclerosis by promoting alternative bile acid-metabolism and sterol excretion

**DOI:** 10.1186/s12933-023-01865-w

**Published:** 2023-06-22

**Authors:** Martin Geiger, Sara Oppi, Stefanie Nusser-Stein, Sarah Costantino, Shafeeq Ahmed Mohammed, Era Gorica, Joanne A. Hoogerland, Christian M. Matter, Ana T. Guillaumon, Frank Ruschitzka, Francesco Paneni, Maaike H. Oosterveer, Sokrates Stein

**Affiliations:** 1 Center for Translational and Experimental Cardiology, University of Zurich, Schlieren, Switzerland; 2 Vascular Diseases Discipline, Clinics Hospital of the University of Campinas, Campinas, Brazil; 3 Department of Pediatrics, Center for Liver Digestive and Metabolic Diseases, University of Groningen, University Medical Center Groningen, Groningen, The Netherlands; 4 Department of Cardiology, University Heart Center Zurich, University Hospital Zurich, CH-8091 Zurich, Switzerland; 5 Department of Research and Education, University Hospital Zurich, CH-8091 Zurich, Switzerland; 6 Department of Laboratory Medicine, University of Groningen, University Medical Center Groningen, Groningen, The Netherlands

**Keywords:** Atherosclerosis, Immunometabolic disease, Mechanism of disease, Nuclear receptor corepressor, Ncor1

## Abstract

**Background:**

The nuclear receptor corepressor 1 (NCOR1) plays an important role in the regulation of gene expression in immunometabolic conditions by connecting chromatin-modifying enzymes, coregulators and transcription factors. NCOR1 has been shown to be involved in cardiometabolic diseases. Recently, we demonstrated that the deletion of macrophage NCOR1 aggravates atherosclerosis by promoting CD36-triggered foam cell formation via PPARG derepression.

**Purpose:**

Since NCOR1 modulates the function of several key regulators involved in hepatic lipid and bile acid metabolism, we hypothesized that its deletion in hepatocytes alters lipid metabolism and atherogenesis.

**Methods:**

To test this hypothesis, we generated hepatocyte-specific *Ncor1* knockout mice on a *Ldlr*-/- background. Besides assessing the progression of the disease in thoracoabdominal aortae *en face*, we analyzed hepatic cholesterol and bile acid metabolism at expression and functional levels.

**Results:**

Our data demonstrate that liver-specific *Ncor1* knockout mice on an atherosclerosis-prone background develop less atherosclerotic lesions than controls. Interestingly, under chow diet, plasma cholesterol levels of liver-specific *Ncor1* knockout mice were slightly higher compared to control, but strongly reduced compared to control mice after feeding them an atherogenic diet for 12 weeks. Moreover, the hepatic cholesterol content was decreased in liver-specific *Ncor1* knockout compared to control mice. Our mechanistic data revealed that NCOR1 reprograms the synthesis of bile acids towards the alternative pathway, which in turn reduce bile hydrophobicity and enhances fecal cholesterol excretion.

**Conclusions:**

Our data suggest that hepatic *Ncor1* deletion in mice decreases atherosclerosis development by reprograming bile acid metabolism and enhancing fecal cholesterol excretion.

**Supplementary Information:**

The online version contains supplementary material available at 10.1186/s12933-023-01865-w.

## Introduction

The liver plays a crucial role in the development of atherosclerosis by regulating immunometabolic processes, such as the expression of pro-inflammatory cytokines and acute phase response proteins, the secretion of VLDL particles, the uptake of cholesterol from the circulation, and biliary cholesterol excretion [1, 2]. An immunometabolic dysregulation in the liver can thus promote the development of several chronic diseases, including Non Alcoholic Fatty Liver Disease (NAFLD) and atherosclerosis. Importantly, NAFLD leads to adverse cardiovascular functions, such as increased oxidative stress and endothelial dysfunction, hypercoagulability, and accelerated atherosclerosis development [3–5].

NCOR1 is a scaffolding protein that forms the basis of a large corepressor complex, including histone deacetylase 3 (HDAC3). NCOR1 suppresses several nuclear receptors, such as liver X receptors (LXRs), peroxisome proliferator-activated receptors (PPARs), and thyroid hormone receptors (THRs) [6, 7]. In macrophages, NCOR1 exerts pro and anti-inflammatory functions [8–11]. In a previous study, we demonstrated that myeloid cell-specific deletion of Ncor1 aggravates atherosclerotic development in atherosclerosis-prone *Ldlr*-deficient mice [12].

Targeted deletions of NCOR1 in the liver, adipose tissue, or muscle alter mitochondrial function, lipid metabolism, and insulin sensitivity, highlighting some of its metabolic functions [8, 13–15]. Previously, Astapova et al. showed that a mouse model with a mutated *Ncor1* lacking 2 nuclear receptor interacting domains (*NCoRΔID*) leads to elevated expression of genes involved in bile acid metabolism as well as canalicular bile salt transport, regulated by thyroid hormone receptor (TR) and liver X receptor (LXR). These mice displayed improved cholesterol tolerance by changing the composition and hydrophobicity of the bile salt pool and diminishing intestinal cholesterol absorption [15]. The authors concluded that the truncated NCOR1 mutant does not exert pro-atherogenic functions in the liver because they did not observe an extra-hepatic accumulation of cholesterol in *NCoRΔID* mice. These mice were fed a high-cholesterol diet (2% cholesterol) for a relatively short time (3 weeks) and were not on an atherosclerosis-prone background [16]. Therefore, the function of hepatic NCOR1 in atherogenesis remains unexplored.

Since NCOR1 transrepresses the function of several nuclear receptors involved in hepatic lipid transport and synthesis, we hypothesized that genetic NCOR1 deletion in the liver alters lipid metabolism and atherosclerosis development. To test this hypothesis, we studied the effects of liver-deficient *Ncor1* mice that were fed a high-cholesterol diet for 12 weeks on atherosclerosis. Our data demonstrated that hepatic deletion of *Ncor1* decreases atherosclerosis development by improving cholesterol tolerance. Hepatic deficiency of *Ncor1* induced changes in the bile acid pool composition, leading increased sterol excretion. Consistently with the findings from Astapova et al., our data suggest that these effects are mediated by increased expression of *Cyp27a1* and *Cyp3a11* in the bile acid (BA) synthesis pathway along with increased expression of canalicular bile salt pump *Abcb11*. The identification of atherogenic targets, such as NCOR1, might lead to the identification of druggable targets and hence to the development of new therapeutic strategies to diminish atherosclerosis disease in hypercholesterolemic patients.

## Methods

### Animal studies and ethics

NCoR1 floxed (NcoR1fl/fl) and (Alb)-Cre mice (Alb-cre Tg/0) were generated using the Cre-loxP system as described before. Briefly, offspring that transmitted the mutated allele, in which the selection marker was excised, and that lost the Flp transgene (NcoR1L2/WT mice) were selected, mated with mouse albumin (Alb)-Cre mice, and then further intercrossed to generate mutant (Alb)-cre Tg/0/-NcoR1L2/L2 mice, which were termed as NcoR1Δhep mice. NcoR1Δhep mice, backcrossed for over 10 generations to C57BL/6J were used in experiments with NcoR1fl/fl used as controls. All these mice lines were on a C57BL/6J background [17]. Animals were kept in small groups (not more than five animals per cage) in single individually-ventilated cages. They had free access to food and water, were maintained at 24  ^o^C, and were kept at a 12-hour light/dark cycle. For atherosclerosis assessment, 8-week-old male mice were fed a high-cholesterol diet (1.25% Cholesterol, sniff Spezialdiäten GmbH no. E15749-34) for 12 weeks. All animal procedures were approved by the Swiss (Canton of Zurich, animal protocol ZH061/16) or by the Dutch Central Committee for Animal Experiments under permit number AVD105002015245 and adhered to guidelines set out in the 2010/63/EU directive.

### Bioinformatics analyses

All raw and/or normalized transcriptomic data are publicly available on Gene Expression Omnibus (GEO) under the accession number GSE49388. Statistical information (positive and negative correlations, significance values) is indicated in the corresponding figure legends.

### Statistics

Statistical analysis was performed with GraphPad Prism (version 6). Data are expressed as scatter plots of individual values with the mean or box plots with the full range of variation (from min to max), the interquartile range and the median. Analysis of *en face* atherosclerotic plaque content was carried out with unpaired, nonparametric *Mann-Whitney U t*-tests. Comparison of differences between two groups of other experiments was assessed using unpaired, parametric two-tailed (multiple) Student’s *t*-tests. Multiple group comparisons were assessed by two-way analysis of variance (ANOVA) and Bonferroni *post hoc t*-tests. Differences under to p < 0.05 were considered statistically significant.

Further methods are specified in the Additonal file 1.

## Results

### Hepatic deletion of Ncor1*** de***creases atherosclerosis development

**Fig. 1:**
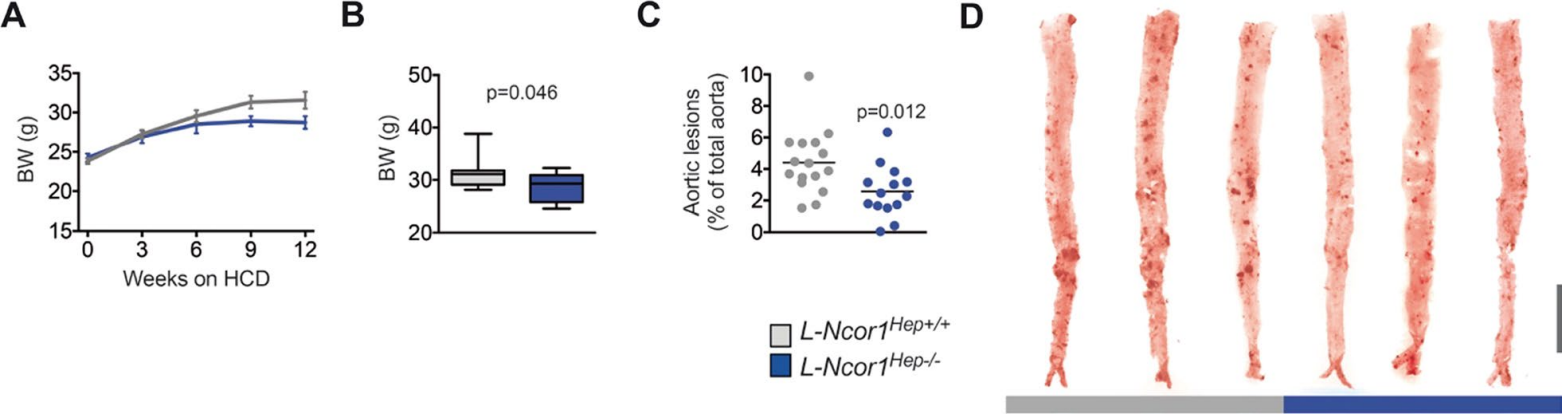
Hepatocyte-specific deletion of *Ncor1* reduces atherosclerosis progression. **A**,** B** Body weight during the high-cholesterol diet (HCD) feeding period of 12 weeks of **A** L-Ncor1^Hep+/+^ and L-Ncor1^Hep−/−^ mice. n = 13 L-Ncor1^Hep+/+^; n = 13 L-Ncor1^Hep−/−^ and **B** L-Ncor1^Hep+/+^ and L-Ncor1^Hep−/−^ mice. n = 13 L-Ncor1^Hep+/+^; n = 13 L-Ncor1^Hep−/−^. **C** Quantification of atherosclerotic plaques in thoracoabdominal aortae of L-Ncor1^Hep+/+^ and L-Ncor1^Hep−/−^ mice. n = 16 L-Ncor1^Hep+/+^; n = 14 L-Ncor1^Hep−/−^. **D** Representative images of Oil-red O stained thoracoabdominal aortae of L-Ncor1^Hep+/+^ and L-Ncor1^Hep−/−^ mice. Scale bar, 5 mm. Data are represented in box plots or scatter plots with means. Displayed P-value relative to L-Ncor1^Hep+/+^, as determined by parametric student’s t-test

To investigate the role of hepatic NCOR1 in atherosclerosis, we generated hepatocyte-specific *Ncor1* knockout mice on an atherosclerosis-prone low-density lipoprotein receptor knockout (*Ldlr*^−/−^) background; further referred to as *L-Ncor1*^Hep−/−^ and control *L-Ncor1*^Hep+/+^ (Additional file 1: Fig. S1 and S2). 8-week-old mice were then placed on a high-cholesterol diet for 12 weeks to accelerate atherogenesis. Body weight was similar to controls at the start of the dietary intervention, *L-Ncor1*^Hep−/−^ mice gained less weight compared to control *L-Ncor1*^Hep+/+^ mice upon high-cholesterol feeding (Fig. 1A, B). To verify whether reduced body weight is a consequence of diminished food intake, we assessed 24 h food intake. Surprisingly, food intake was increased in *Ncor1*^Hep−/−^ compared to control *L-Ncor1*^Hep+/+^ mice (Additional file 1: Fig. S3), suggesting reduced feeding efficacy. Interestingly, upon feeding the mice on a high-cholesterol diet developed less thoraco-abdominal lesions compared to *L-Ncor1*^Hep+/+^ controls (Fig. 1C, D). Our findings highlight a clear-cut phenotype: hepatic deletion of *Ncor1* reduces atherosclerosis progression.

### Hepatocyte-specific *Ncor1* knockouts display reduced plasma and liver cholesterol contents

**Fig. 2:**
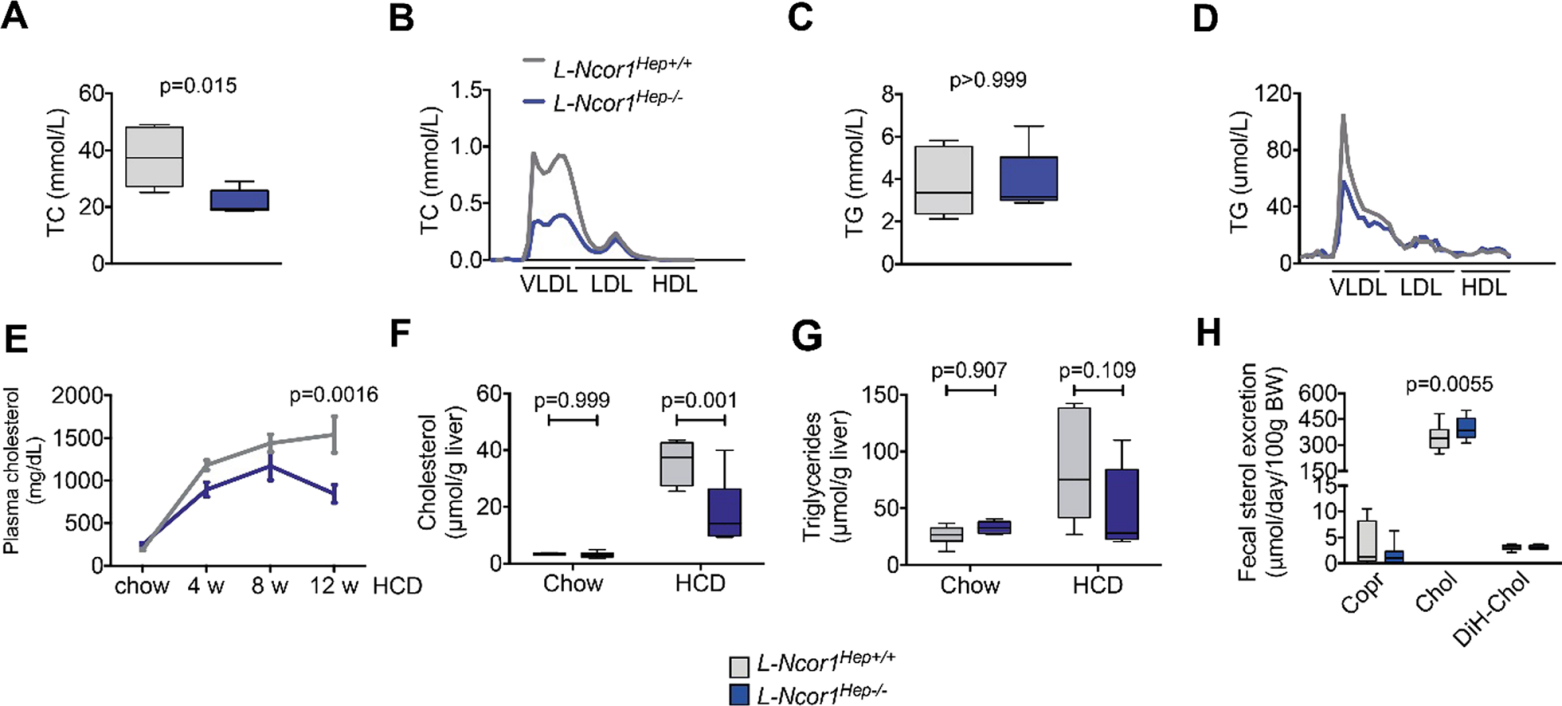
Hepatocyte-specific *Ncor1* knockouts display reduced plasma and liver cholesterol contents. **A** Content of total cholesterol in plasma (TC) in L-Ncor1^Hep+/+^ and L-Ncor1^Hep−/−^ mice n = 5 L-Ncor1^Hep+/+^; n = 5 L-Ncor1^Hep−/−^.** B** Content of lipoprotein subfractions in L-Ncor1^Hep+/+^ and L-Ncor1^Hep−/−^ mice. Pooled samples from 5 to 7 mice per genotype. **C** Content of triglycerides in plasma (TG) in L-Ncor1^Hep+/+^ and L-Ncor1^Hep−/−^ mice n = 5 L-Ncor1^Hep+/+^; n = 5 L-Ncor1^Hep−/−^. **D** Content of lipoprotein subfractions in L-Ncor1^Hep+/+^ and L-Ncor1^Hep−/−^ mice. n = 6 L-Ncor1^Hep+/+^; n = 8 L-Ncor1^Hep−/−^. **E** Plasma cholesterol content during HCD exposure in L-Ncor1^Hep+/+^ and L-Ncor1^Hep−/−^ mice. n = 12 L-Ncor1^Hep+/+^; n = 11 L-Ncor1^Hep−/−^. **F** Liver cholesterol content in L-Ncor1^Hep+/+^ and L-Ncor1^Hep−/−^ mice chow diet and HCD. n = 6 L-Ncor1^Hep+/+^ in chow diet; n = 5 L-Ncor1^Hep+/+^ in HCD; n = 6 L-Ncor1^Hep−/−^ in chow diet; n = 6 L-Ncor1^Hep−/−^ in HCD. **G** Liver triglycerides content in L-Ncor1^Hep+/+^ and L-Ncor1^Hep−/−^ mice chow diet and HCD. n = 6 L-Ncor1^Hep+/+^ in chow diet; n = 5 L-Ncor1^Hep+/+^ in HCD; n = 6 L-Ncor1^Hep−/−^ in chow diet; n = 5 L-Ncor1^Hep−/−^ in HCD. **H** Quantification of fecal sterol excretion in L-Ncor1^Hep+/+^ and L-Ncor1^Hep−/−^ mice; n = 11 L-Ncor1^Hep+/+^ ; n = 11 L-Ncor1^Hep−/−^. p relative to wild-type control. Hepatic *NCor1* deficiency alters the bile acid composition and increases the fecal excretion of sterols. Data are represented in box plots with means. Displayed P-value relative to L-Ncor1^Hep+/+^, as determined by parametric student’s *t*-tests. All data are from mice fed a high-cholesterol diet. Copr: coprostanol, Chol: cholesterol, DiH-Chol: DiH-cholesterol

Consistent with the athero-protective phenotype in mice, we observed that the *L-Ncor1*^Hep−/−^ mice showed lower plasma cholesterol levels compared to *L-Ncor1*^Hep+/+^ controls when exposed to a high-cholesterol diet (Fig. 2A). The difference in total cholesterol and cholesterol subfractions between genotypes was insignificant before the start of the diet (Additional file 1: Fig. S4 A, B). Interestingly, prior to start the high-cholesterol diet we observed slightly increased plasma cholesterol levels in *Ncor1*^Hep−/−^ compared to control *L-Ncor1*^Hep+/+^ mice. The difference in the cholesterol content, especially in VLDL and LDL subfractions, became very clear upon comparison of the lipoprotein profiles in both chow and high-cholesterol fed mice of both genotypes (Additional file 1: Fig. S4C, D). *L-Ncor1*^Hep−/−^ failed to display the same striking rise in VLDL/LDL-cholesterol that is typically observed upon high-cholesterol diet feeding (Fig. 2B). Total plasma triglyceride levels were not changed, although a slight reduction in VLDL-associated triglyceride was observed in *L-Ncor1*^Hep−/−^ compared to controls (Fig. 2C, D). Already after four weeks of high-cholesterol diet feeding *L-Ncor1*^Hep−/−^ mice displayed lower levels of plasma cholesterol compared to control *L-Ncor1*^Hep+/+^ (Fig. 2E).

These findings in *L-Ncor1*^Hep−/−^ mice were intriguing and could be a consequence of differences in dietary cholesterol availability, accumulation of cholesterol in the liver, or a change in intestinal cholesterol (re)absorption and/or excretion. As food intake was rather higher than lower in *L-Ncor1*^Hep−/−^ mice compared to *L-Ncor1*^Hep+/+^ littermates, reduced dietary cholesterol intake, does not explain the plasma cholesterol phenotype. Moreover, hepatic cholesterol content was found to be reduced rather than increased in high-cholesterol fed *L-Ncor1*^Hep−/−^ compared to *L-Ncor1*^Hep+/+^ mice (Fig. 2F), whereas hepatic triglyceride levels were not altered (Fig. 2G). However, *L-Ncor1*^Hep−/−^ mice also showed increased fecal excretion of cholesterol (Fig. 2H).

## Hepatic *NCor1* deficiency alters bile acid composition and increases fecal sterol excretion

To understand the higher fecal excretion observed in knockout mice, we performed a series of in vivo studies to assess cholesterol and bile acid metabolism. Since several nuclear receptors control bile acid metabolism, we quantified the bile flow and its constituents. No difference between.

**Fig. 3:**
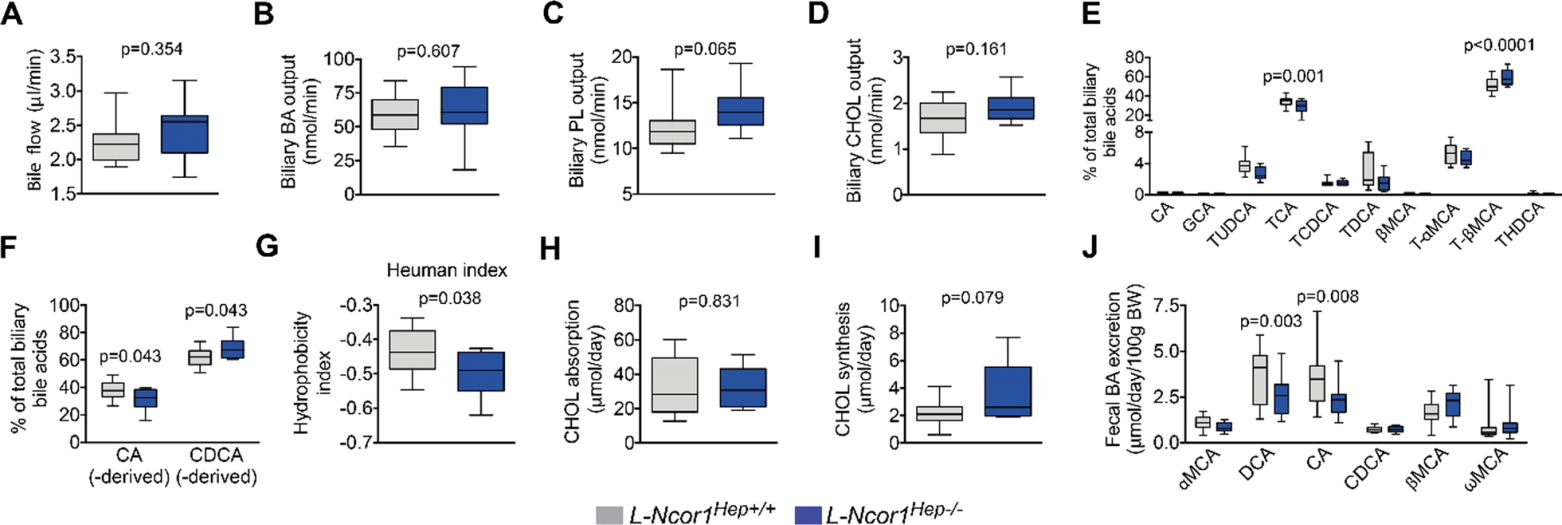
Hepatic *Ncor1* deficiency alters bile acid composition and increases fecal sterol excretion. **A** Bile flow during 30 min during biliary cannulation in L-Ncor1^Hep+/+^ and L-Ncor1^Hep−/−^ mice n = 11 L-Ncor1^Hep+/+^; n = 11 L-Ncor1^Hep−/−^ . **B**–**D** Biliary content of bile acids in L-Ncor1^Hep+/+^ and L-Ncor1^Hep−/−^ mice n = 11 L-Ncor1^Hep+/+^; n = 10 L-Ncor1^Hep−/−^ **E, F** Percent of CA- and CDCA-derived bile acid from the total biliary pool cannulation in L-Ncor1^Hep+/+^ and L-Ncor1^Hep−/−^ mice n = 11 L-Ncor1^Hep+/+^; n = 11 L-Ncor1^Hep−/−^. **G** Bile hydrophobicity as estimated by the Heuman’s method in L-Ncor1^Hep+/+^ and L-Ncor1^Hep−/−^ mice n = 11 L-Ncor1^Hep+/+^; n = 11 L-Ncor1^Hep−/−^ . **H** Cholesterol absorption in L-Ncor1^Hep+/+^ and L-Ncor1^Hep−/−^ mice n = 9 L-Ncor1^Hep+/+^; n = 8 L-Ncor1^Hep−/−^. **I** Cholesterol synthesis in L-Ncor1^Hep+/+^ and L-Ncor1^Hep−/−^ mice n = 9 L-Ncor1^Hep+/+^; n = 8 L-Ncor1^Hep−/−^. **J** Fecal bile acid excretion in L-Ncor1^Hep+/+^ and L-Ncor1^Hep−/−^ mice n = 11 L-Ncor1^Hep+/+^; n = 11 L-Ncor1^Hep−/−^. Data are represented in box plots with means. Displayed P-value relative to L-Ncor1^Hep+/+^, as determined by parametric student’s t-test and ANOVA and Bonferroni’s post hoc *t*-tests for multiple group comparisons. All data are from mice fed a high-cholesterol diet. CA: cholic acid, CDCA: chenodeoxycholic acid, GCA: glycocholic acid, DCA: deoxycholic acid, TUDCA: tauroursodeoxycholic acid, TCA: taurocholic acid, TCDCA: taurochenodeoxycholic acid, TDCA: taurodeoxycholic acid, βMCA: β muricholic acid, T-αMCA: tauro α muricholic acid, T-βMCA: tauro β muricholic acid, THDCA: taurohyodeoxycholic acid, αMCA: α muricholic acid, βMCA: β muricholic acid, ωMCA: ω muricholic acid

*L-Ncor1* ^Hep−/−^ and *L-Ncor1*^Hep+/+^ mice was noted in bile flow as well as of total bile acid concentrations, and biliary phospholipids, and cholesterol levels (Fig. 3A–D). However, we observed a shift in the composition of specific bile acid species (Fig. 3E and Additional file 1: Fig. S5), which in turn led to a decrease of cholic acid (CA)-derived species and a corresponding increase of chenodeoxycholic acid (CDCA)-derived species in *L-Ncor1*^Hep−/−^ compared to *L-Ncor1*^Hep+/+^ mice (Fig. 3F). The different composition of the bile acid species affects the hydrophobicity and related solubilisation efficacy of the bile, which in turn may impact intestinal cholesterol absorption and excretion [18–20] Indeed, we observed that the bile from the *L-Ncor1*^Hep−/−^ mice was less hydrophobic compared to the bile from *L-Ncor1*^Hep+/+^ mice as determined by the Heuman index (Fig. 3G), confirming previous observations with an independent *Ncor1*-deficient mouse model on a non-atherosclerosis prone genetic background [15]. Interestingly, we did not see any changes in the intestinal absorption of cholesterol (Fig. 3H), and no difference in hepatic cholesterol synthesis (Fig. 3I). Fecal bile acid excretion was not altered (Fig. 3J and Additional file 1: Fig. S6).

### Hepatic *Ncor1* deficiency alters expression of genes involved in alternative bile acid synthesis

**Fig. 4:**
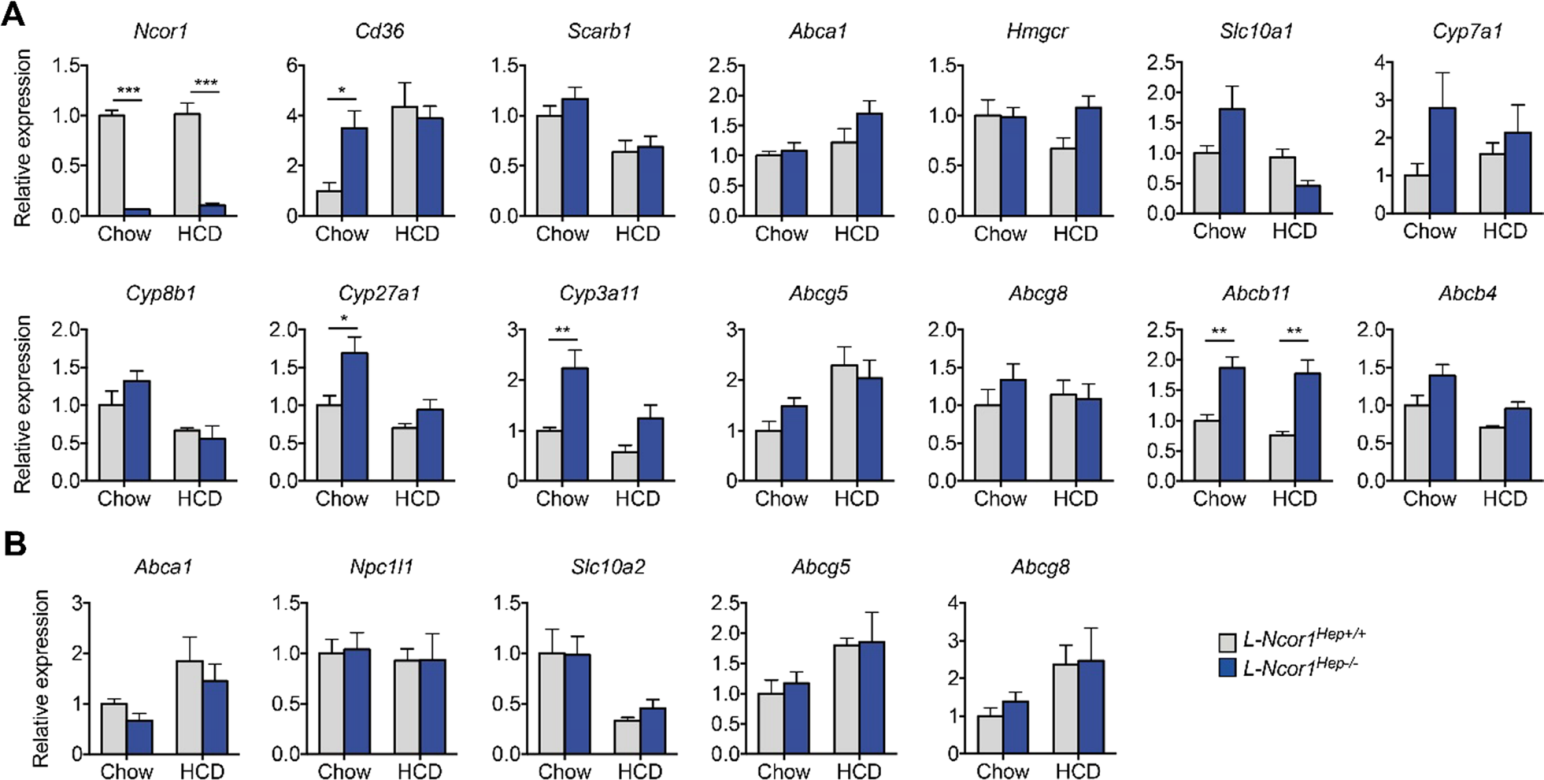
Hepatic *Ncor1* deficiency alters expression of genes involved in alternative bile acid synthesis. **A** The expression of genes regulating bile acid synthesis in the liver of L-Ncor1^Hep+/+^ and L-Ncor1^Hep−/−^ mice in chow and in HCD diet. n = 6 L-Ncor1^Hep+/+^ in chow diet; n = 5 L-Ncor1^Hep+/+^ in HCD; n = 6 L-Ncor1^Hep−/−^ in chow diet; n = 6 L-Ncor1^Hep−/−^ in HCD. **B** The expression of genes regulating lipid transport absorption in the jejunum of male L-Ncor1^Hep+/+^ and L-Ncor1^Hep−/−^ mice in chow and in HCD diet. n = 6 L-Ncor1^Hep+/+^ in chow diet; n = 5 L-Ncor1^Hep+/+^ in HCD; n = 6 L-Ncor1^Hep−/−^ in chow diet; n = 5 L-Ncor1^Hep−/−^ in HCD. Data are represented in bar charts with S.E.M *p < 0.05, **p < 0.01, and ***p < 0.001 relative to L-Ncor1^Hep+/+^, as determined by parametric student’s t-test

To explore the molecular mechanisms driving the changes in bile composition and cholesterol excretion and synthesis, we assessed the expression of various enzymes and transporters in the liver and intestine. *Cyp8b1* and *Cyp7a1*, predominantly involved in the classical pathway of primary bile acid synthesis, were not altered by either genetic background of diet (Fig. 4A). The expression of *Cyp27a1*, a critical enzyme that catalyses the first step in the oxidation of the steroid side chain in the alternative bile acid production pathway, was upregulated in the liver of *L-Ncor1*^Hep−/−^ mice. The upregulation of *Cyp27a1* and *Cyp3a11* genes in *L-Ncor1*^Hep−/−^ mice are primarily responsible for the altered bile composition, therefore changing its hydrophobicity, and increasing the fecal excretion of cholesterol.

Moreover, we found elevated expression levels of *Abcb11* in *L-Ncor1*^Hep−/−^ mice under both, background and dietary conditions (Fig. 4A). The protein encoded by this gene is the central canalicular bile salt export pump. The level of *Slc10a1*, a gene responsible to produce Na^+^-taurocholate co-transporting polypeptide (NTCP), one of the critical bile acid co-transporters that mediates the hepatic uptake of bile acids, remained unaltered (Fig. 4A). The expression of several genes regulating lipid metabolism, including fatty acid and bile acid synthesis, are regulated in a circadian and/or feeding pattern. We thus analyzed several targets at three times of the day: 24 h (Zeitgeber ZT17), 8 h (ZT1) and 16 h (ZT9). Although we observed some trends for changes in PPARγ targets, such as *Cd36*, almost no difference was evident for LXR targets, such as *Abcg5/Abcg8*. Consistent with our findings, we observed the strongest impact on the expression of alternative bile acid synthesis regulators, such as *Cyp27a1* and *Cyp3a11* (Additional file 1: Fig. S7). Regarding the gastrointestinal tract we assessed the expression more specifically in the jejunum. No difference in expression was noted for the major cholesterol transporters in *L-Ncor1*^Hep−/−^ mice (Fig. 4B).

## Discussion

Atherosclerosis is characterized by the accumulation of immune cells, cholesterol-species and other lipids in the intimal space of arteries [21]. One hallmark of the disease is the excessive accumulation of cholesterol in monocyte-derived macrophages within atherosclerotic lesions. The complex pathophysiology is triggered by genetic and environmental risk factors. Importantly, these risk factors converge on various molecular processes, including inflammatory and/or metabolic responses, in diverse organs and the cells within atherosclerotic plaques [22]. Inflammatory and metabolic mediators activate signaling pathways that converge at key transcriptional regulators. These transcriptional regulators coordinate the expression of downstream target genes, and specific transcriptional cofactors can act as central immunometabolic regulators. Some of these factors are involved in inflammation, while others are involved in metabolic functions. Nuclear receptor corepressor 1 (NCOR1) is a central transcription corepressor involved in both processes [23]. In a previous study we demonstrated that macrophage NCOR1 suppresses PPARγ-driven CD36 expression, foam cell formation and atherogenesis [12].

Bile acid synthesis predominantly occurs in hepatocytes and is a pivotal process in the cholesterol catabolism. Cholic acid (CA) and chenodeoxycholic acid (CDCA) are the major primary bile acids synthesized and the classic pathway is initiated by cholesterol 7α-hydroxylase (CYP7A1). On the other hand, the alternative pathway, initiated by sterol 27-hydroxylase (CYP27A1) takes place in the liver as well as other organs [24, 25].

It was previously shown that a truncated NCOR1 mutant (NCoRΔID) that lacks interaction with thyroid hormone receptor (TR) and liver X receptor (LXR) leads to an improvement in serum cholesterol upon high-cholesterol feeding [15]. This study did not explore the physiological relevance of the described effects on atherogenesis and therefore the current work aimed to fill this gap by investigating the role of hepatic NCoR1 in atherogenic conditions.

Our data demonstrate consistent findings as reported by Astapova et al. at the mechanistic level: the hepatic deficiency of *Ncor1* led to an overexpression of the bile acid synthesis genes *Cyp27a1* and *Cyp3a11*, and thus to an induction of alternative bile acid synthesis and less hydrophobic bile. In our atherosclerosis mouse model, deletion of hepatic *Ncor1* led to reduced plasma cholesterol concentrations and diminished development of atherosclerotic lesions in the thoraco-abdominal aortae. The phenotype was associated with reduced bile hydrophobicity and enhanced fecal sterol excretion, whereas intestinal absorption was not altered.

Cyp27A1 is a member of the cytochrome P450 superfamily of enzymes and catalyzes many reactions involved in drug metabolism and synthesis of cholesterol, steroids and other lipids. Since the conversion of cholesterol to bile acids is the major route for removing cholesterol from the body, this protein is important for overall cholesterol homeostasis. Cyp27A1 mutations in humans lead to the development of cerebrotendinous xanthomatosis (CTX) [26]. Surprisingly, Cyp27a1 knockout mice (Cyp27a1^-/-^) do not present a CTX phenotype despite generating a similar global pattern of sterols [27]. Hepatic overexpression of Cyp27a1 in mice leads to a generally mild phenotype [15, 28], thus it likely contributed to the overall phenotype observed in our *Ncor1*-deficient mouse model.

Mouse *Cyp3a11* is an homologous gene of human *CYP3A4*, and is believed to perform similar functions [29]. CYP3A shows evidence that it may participate in the regulation of lipid metabolism, even though it is not fundamental [30, 31]. Previous findings showed that reduced cholesterol intake decreased the expression of *Cyp3a11* to, maintain hepatic cholesterol level, corroborating the role of this enzyme in cholesterol homeostasis [30]. These data are in accordance with the results observed in our study, where the *Cyp3a11* upregulation is likely responsible for the bile acid pool composition and improved cholesterol tolerance.

One limitation of our study is that the data was obtained from a mouse model. Future studies should be carried out in human specimens and cell lines, especially considering the differences in bile acid metabolism and resulting hydrophobicity between mice and man [32].

## Conclusion

Our study demonstrated that hepatic deletion of *Ncor1* reduces atherosclerosis development in *Ldlr* knockout mice. In line with the previous findings, *Ncor1* deletion in hepatocytes led to reduced plasma and liver cholesterol levels. Our data indicate that these changes are secondary to the induction of alternative bile acid synthesis, leading to reduced bile hydrophobicity and improved sterol excretion.

## Supplementary Information

**Additional file 1:**  **Figure S1. **Generation of the atherosclerosis-prone hepatocyte-specific *Ncor1* knockout mouse model. **Figure S2. **Validation of the hepatocyte-specific *Ncor1* knockout mouse model. **Figure S3. **Food intake. 24 hours average food intake in L-Ncor1^Hep+/+^ and L-Ncor1^Hep-/-^mice. n = 11 L-Ncor1^Hep+/+^; n = 12 L-Ncor1^Hep-/-^. **Figure S4. **Hepatocyte-specific *Ncor1* knockouts display a trend for increased plasma cholesterol and triglyceride levels. **A** Plasma total cholesterol concentrations in L-Ncor1^Hep+/+^ and L-Ncor1^Hep-/-^ mice n = 6 L-Ncor1^Hep+/+^; n = 9 L-Ncor1^Hep-/-^. **B** TC levels in lipoprotein subfractions of L-Ncor1^Hep+/+^ and L-Ncor1^Hep-/-^ mice. n = 6 L-Ncor1^Hep+/+^; n = 8 L-Ncor1^Hep-/-^. **C **TC levels in lipoprotein subfractions of L-Ncor1^Hep+/+^mice fed HCD or chow diets. Pooled samples of 5–7 mice per diet group. **D** TC levels in lipoprotein subfractions of L-Ncor1^Hep-/-^mice fed HCD or chow diets. Pooled samples of 5–7 mice per diet group. **Figure S5**. Hepatocyte Ncor1 deficiency alters biliary bile acid composition. Percentage of CA- and CDCA-derived bile acids in bile collected through cannulation in L-Ncor1^Hep+/+^ (WT) and L-Ncor1^Hep-/-^ (KO) mice n = 11 L-Ncor1^Hep+/+^; n = 11 L-Ncor1^Hep-/-^. *p < 0.001 relative to L-Ncor1^Hep+/+^. **Figure S6**. Total fecal bile acid excretion. L-Ncor1^Hep+/+^ and L-Ncor1^Hep-/-^ mice. n = 12 L-Ncor1^Hep+/+^; n = 10 L-Ncor1^Hep-/-^. **Figure S7**. Relative expression of transcripts at the indicated times points: 24h (Zeitgeber ZT 17), 8h (ZT 1) and 16h (ZT 9). L-Ncor1^Hep+/+^ and L-Ncor1^Hep-/-^ mice. n = 3 L-Ncor1^Hep+/+^; n = 3 L-Ncor1^Hep-/-^.

## Data Availability

All the raw data are available upon request.
