# Supplementary material for: Genetic deletion of hepatic NCOR1 protects from atherosclerosis by promoting alternative bile acid-metabolism and sterol excretion
Source: Cardiovasc Diabetol. 2023 Jun 22;22:144. doi: 10.1186/s12933-023-01865-w (PMC10288794; doi:10.1186/s12933-023-01865-w)
Supplement: Supplementary file 1 — Additional file 1: Figure S1. Generation of the atherosclerosis-prone hepatocyte-specific Ncor1 knockout mouse model. Figure S2. Validation of the hepatocyte-specific Ncor1 knockout mouse model. Figure S3. Food intake. 24 hours average food intake in L-Ncor1Hep+/+ and L-Ncor1Hep-/-mice. n = 11 L-Ncor1Hep+/+; n = 12 L-Ncor1Hep-/-. Figure S4. Hepatocyte-specific Ncor1 knockouts display a trend for increased plasma cholesterol and triglyceride levels. A Plasma total cholesterol concentrations in L-Ncor1Hep+/+ and L-Ncor1Hep-/- mice n = 6 L-Ncor1Hep+/+; n = 9 L-Ncor1Hep-/-. B TC levels in lipoprotein subfractions of L-Ncor1Hep+/+ and L-Ncor1Hep-/- mice. n = 6 L-Ncor1Hep+/+; n = 8 L-Ncor1Hep-/-. C TC levels in lipoprotein subfractions of L-Ncor1Hep+/+mice fed HCD or chow diets. Pooled samples of 5–7 mice per diet group. D TC levels in lipoprotein subfractions of L-Ncor1Hep-/-mice fed HCD or chow diets. Pooled samples of 5–7 mice per diet group. Figure S5. Hepatocyte Ncor1 deficiency alters biliary bile acid composition. Percentage of CA- and CDCA-derived bile acids in bile collected through cannulation in L-Ncor1Hep+/+ (WT) and L-Ncor1Hep-/- (KO) mice n = 11 L-Ncor1Hep+/+; n = 11 L-Ncor1Hep-/-. *p < 0.001 relative to L-Ncor1Hep+/+. Figure S6. Total fecal bile acid excretion. L-Ncor1Hep+/+ and L-Ncor1Hep-/- mice. n = 12 L-Ncor1Hep+/+; n = 10 L-Ncor1Hep-/-. Figure S7. Relative expression of transcripts at the indicated times points: 24h (Zeitgeber ZT 17), 8h (ZT 1) and 16h (ZT 9). L-Ncor1Hep+/+ and L-Ncor1Hep-/- mice. n = 3 L-Ncor1Hep+/+; n = 3 L-Ncor1Hep-/-. [file 12933_2023_1865_MOESM1_ESM.docx]

Supplementary Material Online for

**Genetic Deletion of Hepatic NCOR1 Protects from Atherosclerosis by Promoting Alternative Bile Acid Metabolism and Sterol Excretion**

**Supplementary Methods.**

**Animal studies and ethics.** *Ncor1* floxed (*Ncor1*fl/fl)^[1]^ and (Alb)-Cre mice (Alb-cre Tg/0)^[2]^ were generated as described before using the Cre-loxP system. Briefly, offspring that transmitted the mutated allele, in which the selection marker was excised, and that lost the Flp transgene (*Ncor1*L2/WT mice) were selected, mated with mouse albumin (Alb)-Cre mice, and then further intercrossed to generate mutant (Alb)-cre Tg/0/-*Ncor1*1L2/L2 mice, which were termed as *Ncor1*Δhep mice. *Ncor1*Δhep mice, backcrossed for over 10 generations to C57BL/6J were used in experiments with *Ncor1*fl/fl (Alb)-cre negative mice as controls. All these mice lines were on a C57BL/6J background. For atherosclerosis assessment, 8-week-old male mice were fed a high-cholesterol diet (1.25 % Cholesterol, sniff Spezialdiäten GmbH no. E15749-34) for 12 weeks. The animals were single caged for 3 days, and the body weight and total food intake were recorded. At the end of this period, feces were collected for further analyses. To determine fractional cholesterol absorption, mice received an intravenous dose of 0.3 mg cholesterol D5 (Medical Isotopes, Inc, Pelham, NH) dissolved in Intralipid (20%; Fre- senius Kabi, Den Bosch, The Netherlands) and an oral dose of 0.6 mg cholesterol-D7 (Cambridge Isotope Laboratories, Inc, Andover, MA) in medium-chain triglyceride oil 10 days before the end of the experiment. Mice furthermore received 2% sodium ^13^C-acetate via the drinking water 3 days before the end of the experiment to determine cholesterol synthesis rates. At the end of the experiment, mice were anesthetized by intraperitoneal injection of Hypnorm (10 mL/kg; Janssen Pharmaceuticals, Tilburg, The Netherlands) and diazepam (10 mg/kg; Actavis, Baarn, The Netherlands), the bile duct was ligated, the gallbladder was cannulated, and bile was collected for 30 minutes. All animal procedures were approved by the Swiss authorities (Canton of Zurich, animal protocol ZH061/16) or by the Dutch Central Committee for Animal Experiments under permit number AVD105002015245 and adhered to guidelines set out in the 2010/63/EU directive.

**Blood lipid analyses**. Pooled plasma samples were subjected to fast protein liquid chromatography (FPLC) gel filtration using a superose 6 column (GE Healthcare, Little Chalfont, UK) as previously described ^[3,4]^. Total plasma or individual fractions were assayed for cholesterol and triglyceride concentrations using commercially available enzymatic assays (Roche, no. 03039773 190 and 11877771 216). Direct blood measurements of total cholesterol, HDL- and LDL-cholesterol, and total triglycerides of mice on a chow diet was performed using a CardioChek PA Analyzer (Polymer Technology Systems, Indianapolis, USA).

**Hepatic lipids concentrations.** Hepatic lipids were extracted by the method of Folch.^[5]^ Approximately 150 mg of frozen livers was extracted in a chloroform: methanol (2:1) mixture. Following addition of 0.9% NaCl and centrifugation, the upper methanol/water phase was remover, and the organic phase was dried then dissolved in butanol/(Triton X-100:methanol [2:1]) (30:20). Triglyceride and cholesterol contents were quantified using enzymatic assays (Roche).

**Biliary and fecal bile acids and cholesterol concentrations.** Biliary bile acid composition was quantified using liquid chromatography-mass spectrometry; fecal bile acid composition was quantified using capillary GC as described .^[6]^ The hydrophobicity index of biliary bile acids was calculated according to Heuman.^[7]^ Biliary and fecal cholesterol and its derivatives were trimethylsilylated with pyridine, N,O- Bis(trimethylsilyl) trifluoroacetaminde, and trimethyl- chlorosilane (ratio 50:50:1) and quantified by GC.^[8]^

**Intestinal cholesterol absorption and cholesterol synthesis***.* Cholesterol absorption was measured using the plasma dual isotope method as described.^[6]^ In short, cholesterol label enrichment was determined by capillary gas chromatography. Cholesterol was derivatized with N,O-Bis (trimethylsilyl) trifluoroacetamide containing 1% trimethylchlorosilane. Isotope ratios were determined. Cholesterol absorption and synthesis were calculated as described elsewhere.^[9–11]^

**Gene expression analysis**. Tissue and cell RNAs were extracted using TRIZOL (Thermo Fisher, no. 15596026). For RT-qPCR, cDNA was generated using the All-in-One cDNA Synthesis kit (Biotool, no. B24403), and analysed by qPCR using a SYBR Green qPCR Master Mix (Biotool, no. B21202) and the primers listed in the supplementary table S5. Expression data were normalized to housekeeping genes, i.e. Actb, B2m and/or Ppib mRNA levels.

***En face* staining**. *En face* plaque analysis was performed on thoraco-abdominal aortae that were fixed with 4% paraformaldehyde (PFA) over night and then stained with Oil-Red O^[3]^. The quantification of the OilRed O positive area was calculated using Fiji (ImageJ).

**Western blot**. Peritoneal macrophages were lysed in TRIS lysis buffer on ice for 20 minutes (50 mM Tris HCl pH 8.0, 125 mM NaCl, 0.25% NP-40 + protease and phosphatase inhibitor cocktail from Thermo Fisher, no. 88669). The cell lysates were centrifuged 10 minutes at maximum speed and the supernatant was used for protein quantification by Bradford colorimetric protein assay (Amresco, M172). Before running SDSPAGE, protein lysates were boiled for 5 min at 95°C. The following antibodies were used for Western blotting: anti-NCOR1 (Cell Signaling, no. 5948), anti-HSP90 (BD Biosciences no. 610418), anti-CD36 (Cell Signaling, no. 14347), anti-GAPDH (Origene, no. OTI2D9)

**Supplementary References**

1. Yamamoto H, Williams EG, Mouchiroud L, Cantó C, Fan W, Downes M, et al. NCoR1 Is a Conserved Physiological Modulator of Muscle Mass and Oxidative Function. Cell 2011;147(4):827–39.

2. Clausen BE, Burkhardt C, Reith W, Renkawitz R, Förster I. [No title found]. Transgenic Res 1999;8(4):265–77.

3. Stein S, Oosterveer MH, Mataki C, Xu P, Lemos V, Havinga R, et al. SUMOylation-dependent LRH-1/PROX1 interaction promotes atherosclerosis by decreasing hepatic reverse cholesterol transport. Cell Metab 2014;20(4):603–13.

4. Stein S, Lemos V, Xu P, Demagny H, Wang X, Ryu D, et al. Impaired SUMOylation of nuclear receptor LRH-1 promotes nonalcoholic fatty liver disease. J Clin Invest 2017;127(2):583–92.

5. Astapova I, Ramadoss P, Costa-e-Sousa RH, Ye F, Holtz KA, Li Y, et al. Hepatic nuclear corepressor 1 regulates cholesterol absorption through a TRβ1-governed pathway. J Clin Invest 2014;124(5):1976–86.

6. de Boer JF, Schonewille M, Boesjes M, Wolters H, Bloks VW, Bos T, et al. Intestinal Farnesoid X Receptor Controls Transintestinal Cholesterol Excretion in Mice. Gastroenterology 2017;152(5):1126-1138.e6.

7. Heuman DM. Quantitative estimation of the hydrophilic-hydrophobic balance of mixed bile salt solutions. J Lipid Res 1989;30(5):719–30.

8. Gamble W, Vaughan M, Kruth HS, Avigan J. Procedure for determination of free and total cholesterol in micro- or nanogram amounts suitable for studies with cultured cells. J Lipid Res 1978;19(8):1068–70.

9. van der Veen JN, van Dijk TH, Vrins CLJ, van Meer H, Havinga R, Bijsterveld K, et al. Activation of the liver X receptor stimulates trans-intestinal excretion of plasma cholesterol. J Biol Chem 2009;284(29):19211–9.

10. Brufau G, Kuipers F, Lin Y, Trautwein EA, Groen AK. A reappraisal of the mechanism by which plant sterols promote neutral sterol loss in mice. PloS One 2011;6(6):e21576.

11. Brufau G, Groen AK. Characterization of Whole Body Cholesterol Fluxes in the Mouse. Curr Protoc Mouse Biol 2011;1(4):413–27.

**Supplementary Figures.**

**
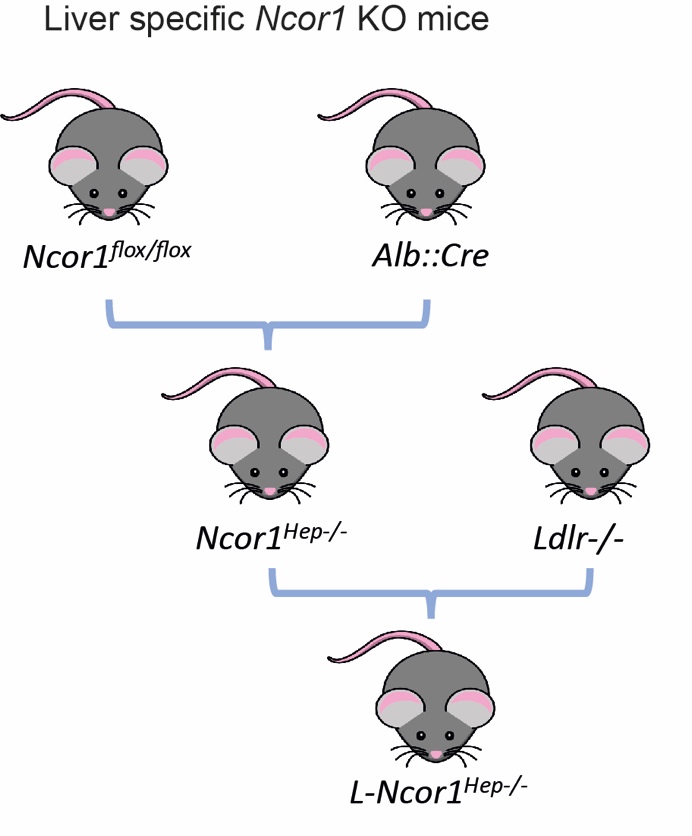
**

**Supplementary Figure S1.** Generation of the atherosclerosis-prone hepatocyte-specific *Ncor1* knockout mouse model


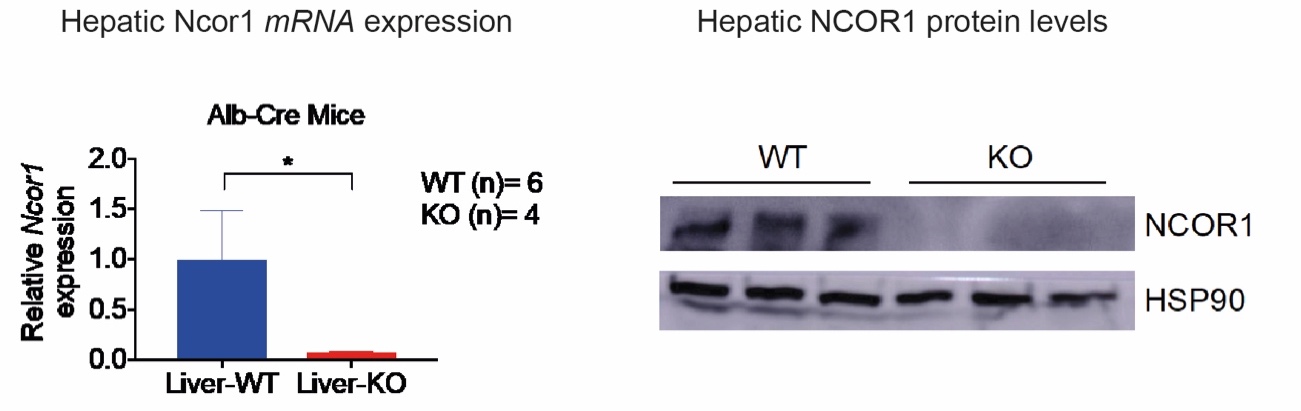


**Supplementary Figure S2.** Validation of the hepatocyte-specific *Ncor1* knockout mouse model.


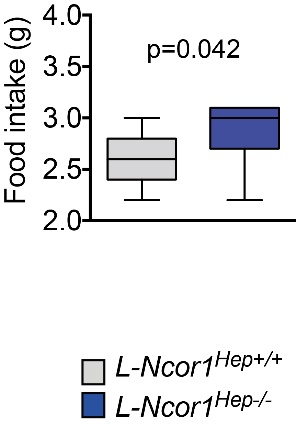


**Supplementary Figure S3. Food intake.** 24 hours average food intake in L-Ncor1^Hep+/+^ and L-Ncor1^Hep-/-^ mice. n= 11 L-Ncor1^Hep+/+^; n= 12 L-Ncor1^Hep-/-^**.**


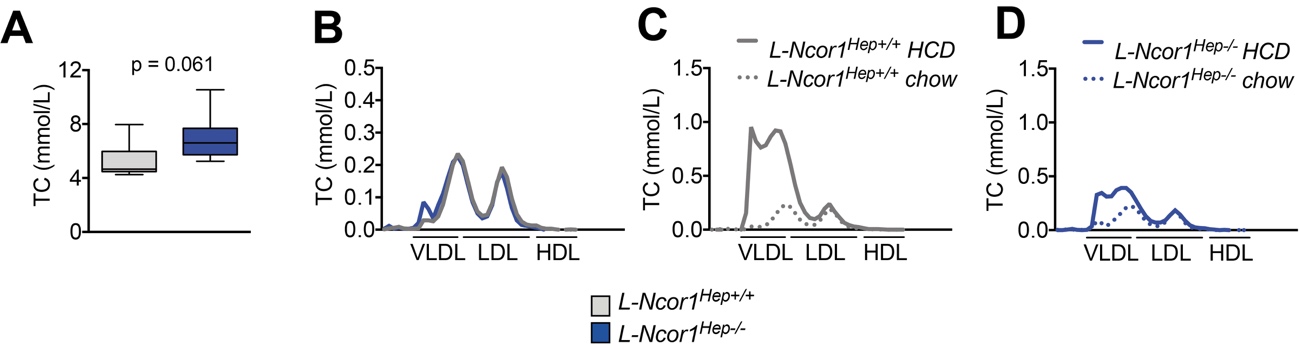


**Supplementary Figure S4. Hepatocyte-specific *Ncor1* knockouts display a trend for increased plasma cholesterol and triglyceride levels.** (**A**) Plasma total cholesterol (TC) concentrations in L-Ncor1^Hep+/+^ and L-Ncor1^Hep-/-^ mice n= 6 L-Ncor1^Hep+/+^; n= 9 L-Ncor1^Hep-/-^ **(B)** TC levels in lipoprotein subfractions of L-Ncor1^Hep+/+^ and L-Ncor1^Hep-/-^ mice. n= 6 L-Ncor1^Hep+/+^; n= 8 L-Ncor1^Hep-/-^. **(C)** TC levels in lipoprotein subfractions of L-Ncor1^Hep+/+^ mice fed HCD or chow diets. Pooled samples of 5-7 mice per diet group. (D) TC levels in lipoprotein subfractions of L-Ncor1^Hep-/-^ mice fed HCD or chow diets. Pooled samples of 5-7 mice per diet group.


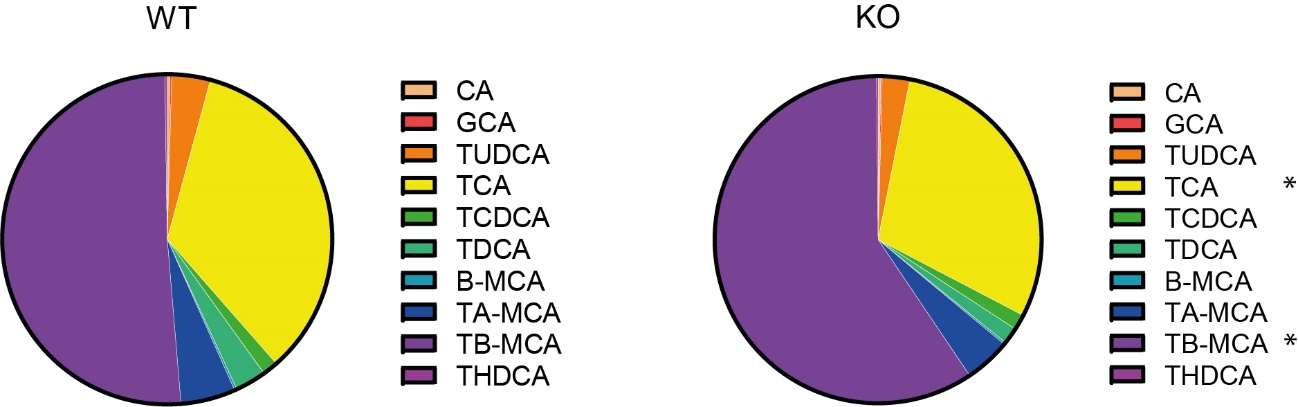


**Supplementary Figure S5. Hepatocyte *Ncor1* deficiency alters biliary bile acid composition.** Percentage of CA- and CDCA-derived bile acids in bile collected through cannulation in L-Ncor1^Hep+/+^ (WT) and L-Ncor1^Hep-/-^ (KO) mice n= 11 L-Ncor1^Hep+/+^; n= 11 L-Ncor1^Hep-/-^. *p<0.001 relative to L-Ncor1^Hep+/+^.


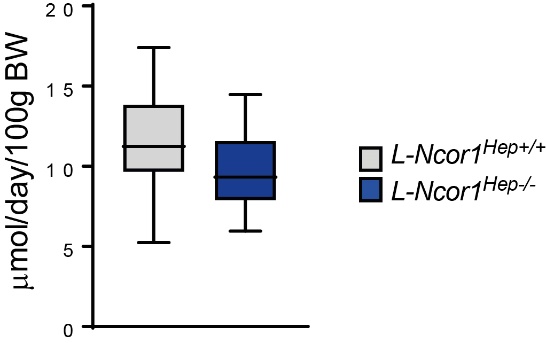


**Supplementary Figure S6. Total fecal bile acid excretion.** L-Ncor1^Hep+/+^ and L-Ncor1^Hep-/-^ mice. n= 12 L-Ncor1^Hep+/+^; n= 10 L-Ncor1^Hep-/-^**.**


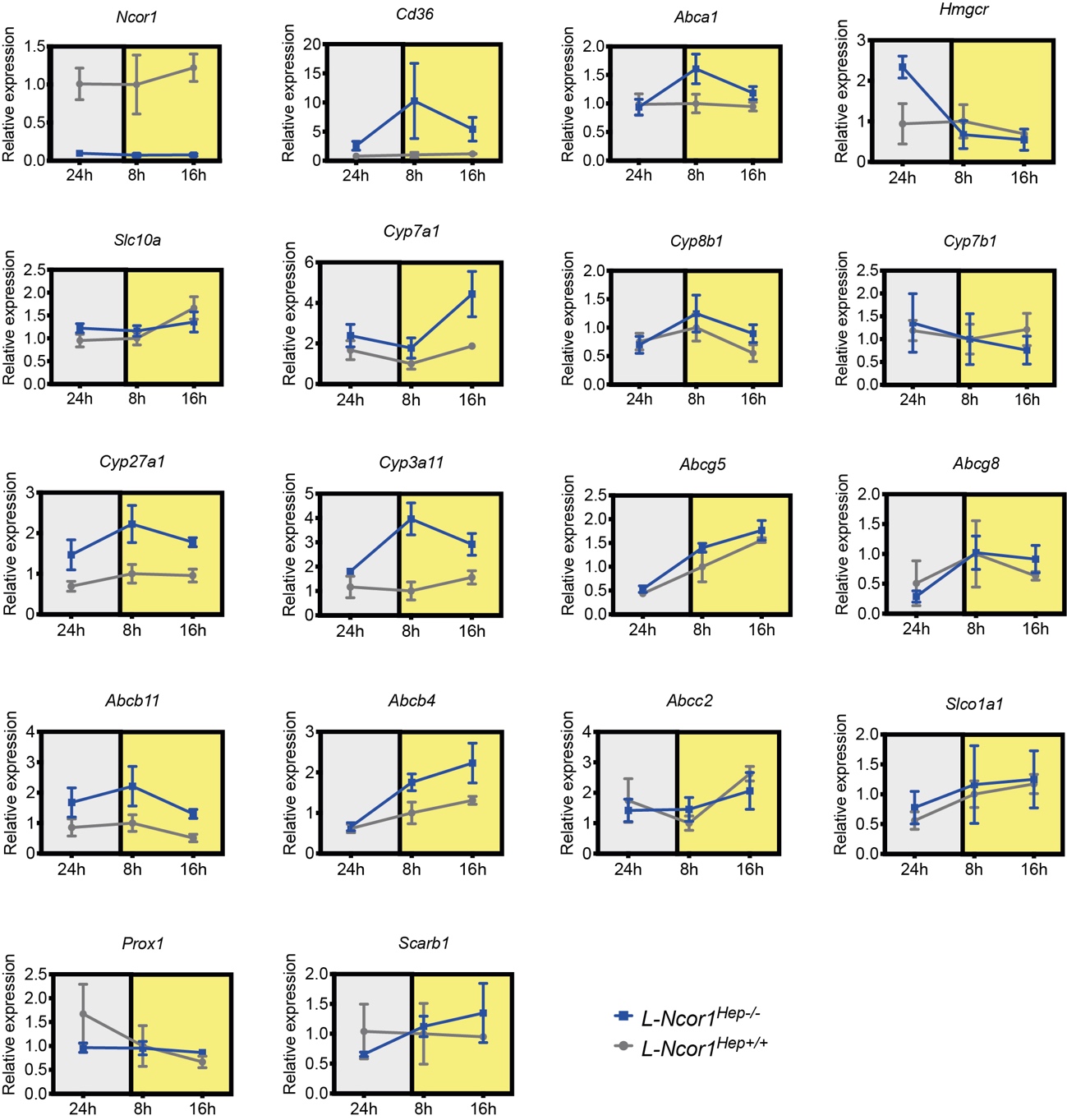


**Supplementary Figure S7. Relative expression of transcripts at three times of the day.** L-Ncor1^Hep+/+^ and L-Ncor1^Hep-/-^ mice. n= 3 L-Ncor1^Hep+/+^; n= 3 L-Ncor1^Hep-/-^**.**

1. **Supplementary Tables.**

**Supplementary Table S1. List of mouse primers used for qPCR**

| Gene | Forward (5’ to 3’ sequence) | Reverse (5’ to 3’ sequence) |
| --- | --- | --- |
| *Ncor1* | CTGGTCTTTCAGCCACCATT | CCTTCATTGGATCCTCCATC |
| *Cd36* | TCCTATTGGCCAAGCTATTGCG | CACGGGGATTCCTTTAAGGTCG |
| *Scarb1* | ACACCCGAATCCTCGCTGGAAT | CCGTTGGCAAACAGAGTATCGG |
| *Abca1* | GGAGCCTTTGTGGAACTCTTCC | CGCTCTCTTCAGCCACTTTGAG |
| *Hmgcr* | GCTCGTCTACAGAAACTCCACG | GCTTCAGCAGTGCTTTCTCCGT |
| *Slc10a1* | CCTGATGCCTTTCACTGGCTTC | GGATGGTAGAACAGAGTTGGACG |
| *Cyp7a1* | ATCAAAGAGCGCTGTCTGGGT | GCGTTAGATATCCGGCTTCAAAC |
| *Cyp8b1* | TTATTCGGCTACACCAAGGACA | CAAATCGACGGAACTTCCTGA |
| *Cyp27a1* | TCAGGAGACCATCGGCACCTTT | CCAGTCACTTCCTTGTGCAAGG |
| *Cyp3a11* | ACAGCACTGGTCAGAGCCTGAA | GAGAGCAAACCTCATGCCAAGG |
| *Abcg5* | TGCCATCCTGACTTACGGAGAG | CTGCTTTGGGTGTCCACTGATG |
| *Abcg8* | GGTCCTTCTGATGACATCTGGC | CGTCTGTCGATGCTGGTCAAGT |
| *Abcb11* | CCTTGGTAGAGAAGAGGCGACA | ATGGCTACCCTTTGCTTCTGCC |
| *Abcb4* | GGAACAAGGAAGTCACAGTGAGC | CAGCAGCCTTTTCGTCACTTAGC |
| *Npc1l1* | ATCGCACTACCATCCAGGACCT | CCCAGAGTAGCCTTGGAATCCA |
| *Slc10a2* | GGTTTCTTCCTGGCTAGACTAGC | GGAAGGTGAACACCAGGTTGAG |
